# Platelets and *Escherichia coli*: A Complex Interaction

**DOI:** 10.3390/biomedicines10071636

**Published:** 2022-07-07

**Authors:** Amina Ezzeroug Ezzraimi, Nadji Hannachi, Antoine Mariotti, Jean-Marc Rolain, Laurence Camoin-Jau

**Affiliations:** 1 IRD, APHM, MEPHI, IHU Méditerranée Infection, Aix Marseille Université, 19-21 Boulevard Jean Moulin, 13005 Marseille, France; amina.ezzeroug.ezzraimi@gmail.com (A.E.E.); n_adji07@live.fr (N.H.); antoine-julien.mariotti@ap-hm.fr (A.M.); jean-marc.rolain@univ-amu.fr (J.-M.R.); 2 IHU Méditerranée Infection, Boulevard Jean Moulin, 13385 Marseille, France; 3 Département de Pharmacie, Faculté de Médecine, Université Ferhat Abbas Sétif I, Sétif 19000, Algeria; 4 Hematology Department, Timone Hospital, APHM, Boulevard Jean Moulin, 13005 Marseille, France

**Keywords:** *Escherichia coli*, platelets, LPS, PMP

## Abstract

Apart from their involvement in hemostasis, platelets have been recognized for their contribution to inflammation and defense against microbial agents. The interaction between platelets and bacteria has been well studied in the model of *Staphylococcus* and *Streptococcus* but little described in Gram-negative bacteria, especially *Escherichia coli*. Being involved in the hemolytic uremic syndrome as well as sepsis, it is important to study the mechanisms of interaction between platelets and *E. coli*. Results of the published studies are heterogeneous. It appears that some strains interact with platelets through the toll-like receptor-4 (TLR-4) and others through the Fc gamma glycoprotein. *E. coli* mainly uses lipopolysaccharide (LPS) to activate platelets and cause the release of antibacterial molecules, but this is not the case for all strains. In this review, we describe the different mechanisms developed in previous studies, focusing on this heterogeneity of responses that may depend on several factors; mainly, the strain studied, the structure of the LPS and the platelet form used in the studies. We can hypothesize that the structure of O-antigen and an eventual resistance to antibiotics might explain this difference.

## 1. Introduction

If platelets were exclusively associated with hemostasis and wound healing for a long time, it is now recognized that they play a key role in the fight against infection. Indeed, platelets share a structure and functional characteristics with immune cells, especially of the myeloid lineage. On their outer membranes, platelets express a set of receptors that allow the recognition of pathogens, making them immune sentinels [1]. During the activation process, platelets quickly change from quiescent discoid forms to amoeboid cells that move to sites of infection.

Studies have shown that a significant number of bacterial species responsible for infections in humans can interact with platelets, causing differences in platelet activation and aggregation. It has been reported that *Staphylococcus aureus* and *Streptococcus pyogenes* rapidly attached to and aggregated on human platelets in vitro [2,3], whereas *Escherichia coli* and *Enterococcus faecalis* caused much slower platelet aggregation. In vitro studies have shown that some strains of bacterial species such as *Fusobacterium, Listeria, Mycobacterium, Pseudomonas, Salmonella* and *Yersinia* are able to induce platelet activation and aggregation [4]. However, the effects obtained depend on the bacterial strain, the platelet-to-bacteria ratio and the inter-individual platelet variability. Differences in this interaction lead to differences in the platelet activation and aggregation process, which likely influences host defense against pathogens. In general, the events associated with the interaction between platelets and bacteria proceed through distinct and progressive phases: direct contact, morphogenesis from discoid to amoeboid form, initial aggregation, and irreversible aggregation. Platelets can be activated by direct contact between platelet receptors and bacterial surface protein, and also by bacterial secreted molecules such as toxins, or indirectly activated via a plasma protein which acts as a bridge between the platelet and the bacteria [3], thus involving platelet receptors FcγRIIA, glycoprotein (GP) αIIbβ3, GPIbα, toll-like receptors, and complement [5].

In response to an activating stimulus, such as bacteria, platelets release molecules which, in addition to their role in the hemostatic process, have been shown to have an antimicrobial effect, and which are today grouped under the name of platelet microbicidal peptides (PMPs) [4].

The mechanisms of interaction between platelets and bacteria have been widely described as in the case of *Staphylococcus aureus* [2,6,7]. On the other hand, they are less detailed in Gram-negative, especially *E. coli*. The objective of this review is to summarize the available data on the mechanisms of interaction between *E. coli* and platelets and their consequences.

## 2. *Escherichia coli* Pathovars

**Figure 1:**
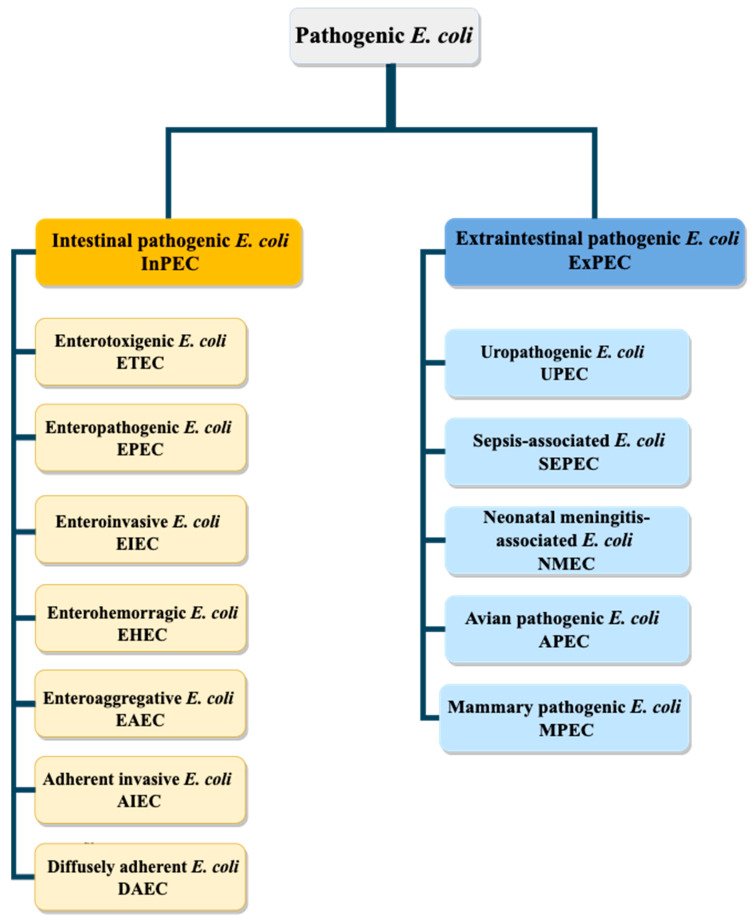
Diagram of pathogenic *E. coli* strains. *E. coli* pathogenic strains can be divided into two groups: extraintestinal pathogenic *E. coli* (ExPEC) and intraintestinal pathogenic *E. coli* (InPEC).

*E. coli* is a commensal bacterium of the gastrointestinal tract of humans, other mammals and birds as well as cold-blooded animals. There are several pathogenic strains which can cause clinical syndromes such as diarrhea, enteric infections, urinary tract infections, meningitis and septicemia, which represent significant clinical cases and cause thousands of deaths per year. The pathogenicity of strains can be explained by different virulence mechanisms such as adhesin expression, toxin secretion, iron acquisition factors, lipopolysaccharide structure, presence of polysaccharide capsules and invasin expression [8,9]. Based on these characteristics, pathogenic *E. coli* can be classified into two groups (Figure 1): intestinal pathogenic *E. coli* (InPEC) and extra-intestinal pathogenic *E. coli* (ExPEC). Each of these two groups contains different subgroups, causing various problems [10].

Despite the ability of extraintestinal pathogenic *E. coli* (ExPEC) group strains to be associated with infections in the intestinal tract, their virulence is significantly higher when they colonize tissues outside the intestine [11,12]. ExPEC themselves can be grouped into five categories; uropathogenic *E. coli* (UPEC), sepsis-associated *E. coli* (SEPEC), neonatal meningitis-associated *E. coli* (NMEC), avian pathogenic *E. coli* (APEC) and *E. coli* mammary pathogenic *E. coli* (MPEC) [8,13].

As for intestinal pathogenic *E. coli* (InPEC), they can be grouped into seven categories: enterotoxigenic *E. coli* (ETEC), enteropathogenic *E. coli* (EPEC), entero-invasive *E. coli* (EIEC), enterohemorrhagic *E. coli* (EHEC), enteroaggregative *E. coli* (EAEC), adherent invasive *E. coli* (AIEC) and diffuse adherent *E. coli* (DAEC) [8,13].

EHEC strains are the most studied, due to their ability to strongly colonize the mucosa of the distal ileum and the colon and to induce so-called "attachment and effacement" lesions of enterocytes via a protein (intimin) encoded by the *eae* gene [14,15]. They are characterized by their somatic antigen O and their flagellar antigen H. The serotypes most involved in epidemics belong to serotypes O26: H11, O103: H2, O111: H8, O145: H28 and O157: H7 [16,17]. EHEC group can cause mild watery diarrhea and hemorrhagic colitis that can progress to severe forms, such as hemolytic-uremic syndrome (HUS), mainly in young children, or thrombotic microangiopathy (TMA) in adults. EHEC, more specifically the Shiga toxin-producing strain *E. coli* STEC, can also release toxins such as Shiga toxins (Stx), inducing damage to the vascular endothelium, mainly intestinal, renal and cerebral, whose *stx* genes have been transferred by bacteriophages [18].

## 3. Mechanism of Interaction between Platelets and *E. coli*

### 3.1. Platelet Receptors

In this part we describe two main platelet receptors: the toll-like receptors (TLRs) and Fc receptors that are involved in interactions with *E coli.*

TLRs belong to a family of receptors that recognize molecular pattern recognition molecules associated with pathogens. The structure of TLRs is characteristic of type I membrane proteins with a transmembrane domain which spans the membrane and connects to an extracellular domain [19]. TLRs play a crucial role in the early innate immune response to pathogens, as the primary defense system against microbial infection.

The interaction of TLRs with microbial products leads to activation of multiple intracellular signaling pathways, which are generally through a MyD88-dependent pathway leading to the stimulation and secretion of inflammatory cytokines, and a Toll/IL-1R domain-containing adaptor inducing IFN-beta (TRIF)-dependent pathway that causes Interferon-β stimulation and dendritic cell maturation [20].

Platelets express TLRs; this highlights the role of platelets in the innate immune response. TLRs 1, 2 and 6 are moderately expressed [21,22,23]. The role of TLR2 has been studied in the activation of the platelet response to *Streptococcus pneumoniae,* which induces platelet aggregation and adenosine diphosphate (ADP) release in both a TLR2- and a TLR4-dependent manner [24,25]. TLR3 is very weakly expressed at the cell surface and intracellular level. Agonizing TLR3-platelet receptors would induce heterogeneous effects [26,27]. TLR7, TLR5, 8 and 10 represent the least studied receptors [28].

Regarding *E. coli*, TLR4 represents the most studied platelet receptor involved in the interaction with this bacterium. The presence of TLR4 was first identified on mouse and human platelets using flow cytometry [29]. As we are specifically interested in the interaction between platelets and *E. coli*, we focused on the TLR4 which is involved in this interaction. LPS stimulates platelet secretion and aggregation requiring the TLR4/MyD88 complex [30,31]. Andonegui et al. were the first to demonstrate that *E. coli* LPS-induced thrombocytopenia was dependent on platelet TLR4. Indeed, thrombocytopenia was observed after administration of LPS only in wild-type mice, not in TLR4-deficient mice. Similarly, an accumulation of platelets in the lungs was observed only in wild-type mice [29]. TLR4 also plays a role in TNF-alpha production after LPS administration in a mouse model. This clearly highlights the role of TLR4 in the process of LPS-induced thrombocytopenia. However, the mechanism by which platelets contribute to increased TNF-alpha levels is still unclear [32].

The strain and concentration of LPS used in these in vitro models are also important variables to consider when analyzing platelet activation by LPS. Human platelets may respond differently to various bacterial LPS via TLR4 engagement, resulting in distinct cytokine secretory profiles with different potency depending on the strains triggering platelet responses [33].

Apart from TLR class receptors, FcγRIIA also plays a key role in *E. coli* and platelet interactions. It is a Type I transmembrane protein of approximately 40 kDa and consists of two extracellular Ig-like domains, a single transmembrane domain and a cytoplasmic tail that bears an immunoreceptor tyrosine-based activation motif (ITAM) domain with dual YXXL amino acid consensus sequences. These receptors are found in platelets, neutrophils and monocytes [34].

Human platelets represent the richest source of FcγRIIA in the body, containing 1000 to 4000 copies of this type of receptor [35]. Platelets, through the FcγRIIA can coat opsonized entities such as bacteria, which leads to platelet activation and the secretion of multiple molecules in order to respond against pathogens [5]. It is through the second Ig-like domain that immunoglobulins bind to FcγRIIA [34]. FcγRIIA is also implicated in signaling being an adapter protein for the platelet integrin αIIbβ3 [34]. Arman et al. have shown that, in addition to the role of FcγRIIA in the initiation of platelet activation after having been involved in the oppsonization of bacteria, the secretion of alpha and dense granules dependent on FcγRIIA and integrin alpha IIb-beta 3 (αIIbβ3) is also important for triggering bacterial aggregation [36]. αIIbβ3, in turn, is also able to stimulate platelet aggregation after being bound to soluble fibrinogen [34]. Regarding *E. coli*, the role of FcγRIIA in platelet activation and aggregation (Table 1A) is linked to conflicting results that will be further detailed below.

### 3.2. E. coli Products

LPS is an important structural component of the outer membrane of Gram-negative bacteria and one of the most studied immunostimulatory elements of bacteria. They can induce systemic inflammation and sepsis if excessive signals occur. LPS consists of three main components: lipid A, the core oligosaccharide which can be divided into an external and an inner part, and thus the O-antigen polysaccharide. Two types of LPS are distinct: smooth and rough LPS. Smooth LPS (S-LPS) contains an O-antigen polysaccharide, additionally to lipid A and core oligosaccharide. In contrast, rough LPS (R-LPS) is deficient in an O-antigen. It has been found that numerous clinical Gram-negative bacteria have S-LPS [37]. LPS can interact with host proteins, including LPS-binding protein, CD14, MD-2 and TLR4 [38]. Platelet activation stimulated by LPS also includes the triggering of pro-inflammatory response, such as the production of cytokine Inteleukin-1 beta, which in turn activates endothelial cells and platelets and participates in tissue damage related to bacterial invasion [27].

Studies have focused on the interaction between LPS from EHEC strains and platelets, with contradictory results. Indeed, some of them have shown that pure LPS from *E. coli* strains are able to induce platelet activation, while others could not show this effect (Table 1B). Interestingly, LPSs are detected on platelet surfaces of children with EHEC-associated HUS, even before HUS developed, but not from children with uncomplicated EHEC infection [39].

Since there are also *E. coli* that secrete the shiga toxin (STEC), it is a well-studied bacterial product in the stimulation of platelet phenomena. Shiga toxin (Stx) is one of the most potent bacterial toxins known. The microbiologist Kiyoshi Shiga was the first to identify the bacterial origin of dysentery caused by *Shigella dysenteriae* in 1897. In 1977, a group of *E. coli* isolates was discovered by Konowalchuk that produce a factor capable of killing vero cells in culture, which is why they were named verotoxin-producing *E. coli* (VTEC) [40]. Shiga toxins (Stx), and verotoxins (VT), are a family of structurally and functionally related cytotoxic proteins produced by enteric pathogens such as Shigella dysenteriae type 1 and STEC.

The Shiga toxin family, a group of structurally and functionally related exotoxins, includes the Shiga toxin of *S. dysenteriae* serotype 1 and the Shiga toxins produced by EHEC. The Shiga toxin 1 produced by *E. coli* (Stx1) is almost identical to the family of Shiga toxin, differing by a single amino acid in the catalytic A subunit of the toxin. STEC (Shiga toxin-producing *E. coli)* can produce Stx1 variants (Stx1 and Stx1c), Stx2 variants (Stx2, Stx2c, Stx2d, Stx2e, Stx2f) or variants of both in a range of combinations [41]. Stx1 and Stx2 are genetically and even immunologically distinct and they share 55–60% genetic and amino acid identity, which is one 32 kDa A subunit and five identical 7.7 kDa B subunits [42,43,44]. Stx1 and 2 differ also in their localization. Stx1 is located in the periplasmic space of the bacterial cell, while Stx2 is in the extracellular fraction [45]. Stx1 is very similar to the Shiga toxin found in *Shigella dysenteriae* type 1. However, it has been demonstrated that Stx2 is 400 times more toxic than Stx1 in a murine infection model [46]. On the other hand, Stx2 is known to be more involved in complications of HUS than Stx1 [47], probably due to the fact that its A1 subunit has a higher affinity for ribosomes and a higher catalyzing potency than A1 subunit of Stx1, which makes it more cytotoxic than Stx1 [48].

Thrombocytopenia is one of the major clinical manifestations of HUS and is associated with the presence of Shiga toxin. Platelets are consumed during the formation of microthrombi and circulating platelets are degranulated [49]. Several studies describe potential interactions of Stx with platelets. Culture filtrates from Stx1-producing *E. coli* can cause platelet aggregation [50]. In parallel, Stx can bind directly to platelets. The toxin is apparently internalized in platelets after incubation, which leads to platelet aggregation. Morphological changes in platelets have been described that may contribute to the prothrombotic state seen in HUS [51], with Shiga toxin bound to activated platelets rather than to resting platelets [52].

### 3.3. Platelets—E. coli Interaction Consequences

The mechanism of platelet activation by Gram-negative bacteria, particularly *E. coli,* has been less studied than Gram-positive bacteria. Recent studies have shown that platelet activation and aggregation by *E. coli* are mainly dependent on the strain and the different receptors involved [53,54,55,56].

To our knowledge, only four studies have investigated the effect of *E. coli* strains on platelets (Table 1A) [53,54,55,56]. Among these studies, only Watson's work used non-EHEC clinical strains. They have demonstrated that platelet activation by the neuropathogenic and uropathogenic *E. coli* strains, CFT073 and RS218, is dependent on FcγRIIA via strain-bound plasma IgG [56]. In contrast, Fejes et al. using laboratory strains (K12 and 018:K1) obtained a different result depending on the strain. Indeed, only the non-pathogenic strain K12 induced platelet activation and granular secretion by a mechanism dependent on platelet glycoprotein IIb IIIa. Two hypotheses would explain this result. The expression of rough LPS, by strain K12, which would induce a more important cellular activation than the smooth LPS of strain O18 and the resistance to the complement of strain O18 could explain this difference of behaviour [53].

Works on EHEC strains have shown that the strains tested induce platelet activation and/or aggregation [54,55]. However, the mechanisms involved differ between the stages of haemostasis studied. Indeed, enterohemorrhagic the *E. coli* O111 strain induces platelet activation as evidenced by the expression of P-selectin and tissue factor. This process is TLR-4 dependent but independent of FcγRIIA [55]. In addition, Moriaty et al. were interested in platelet aggregation induced by the *E. coli* O157 strain. They demonstrated that platelet aggregation observed was TLR4- and FcγRIIA-dependent [55]. It could therefore be hypothesized that TLR4 is involved in activation phenomena. On the other hand, FcγRIIA would be necessary for platelet aggregation mechanisms.

The other studies looked specifically at the effect of pure LPS (Table 1B). The response of platelets to different LPS serotypes is not homogeneous; sometimes it is contradictory between studies, making the role of LPS in platelet activation and aggregation complex and dependent on several factors. LPS from *E. coli* O157 and other EHEC strains induced platelet binding and activation through the expression of GPIIb/IIIa, CD40L and fibrinogen binding. Activation was TLR4-dependent [39]. LPS from O111:B4, O55:B5 and O127:B8 caused platelet activation requiring TLR4/MD2, CD14 and MyD88 and induced platelet aggregation [31]. However, in another study, no platelet activation or aggregation was found with the same strain of *E. coli* O111:B4 LPS [57].

Ståhl et al. compared the effect of four pure LPSs of different serotypes (O157:H7, O103:H2, O111:HN, O121:H19) on platelet activation. Among the LPS tested, only O157:H7 LPS was able to induce higher platelet activation, which could be explained by the fact that there are differences in the LPS O-chain. LPS binding was abrogated by anti-TLR4 and anti-CD62P antibodies, suggesting that these receptors act in concert to bind LPS. However, a steric interaction between the antibodies and their receptors could not be excluded, due to the proximity of TLR4 and CD62 to the platelet surface [39].

In an interesting study, Moriarty et al. compared the effects of *E. coli* O157:H7 and LPS serotype from this strain. Only the whole bacterium induced platelet aggregation; no effect is observed with pure LPS. They suggested that the aggregation was not mediated by a direct interaction, but was enhanced by the action of the complement system [55].

**Table 1:** Summary of *E. coli* strains and *E. coli* pure LPS effect on platelets.

(A) *E. coli* Strain Effect on Platelet Activation and Aggregation					
LPS/*E. coli* Strain	Strain Origin	Platelet Form	Reported Interaction Result	Platelet Receptor Involved	Reference
CFT073 RS218	Pyelonephritis and bacteremia Neonatal meningitis	PRP	Activation and aggregation	FcγRIIA and αIIbβ3	[56]
K12	Non pathogen	PRP	Activation and dense granule release	GPIIb/IIIa	[53]
O18:K1	Pathogen	PRP	-	-	[53]
O111	EHEC	PRP	Activation	TLR4	[54]
O157:H7	EHEC	PRP	Aggregation	αIIbβ3/ Fc	[55]
**(B) *E. coli* LPS Effect on Platelet Activation and Aggregation**					
O111:B4	EHEC	PRP	-	-	[57]
O111:B4, O55:B5 and O127:B8	EHEC	PRP	Activation Alpha and dense granule secretion	CD14, TLR4/MD2 and MyD88	[31]
O157:H7	EHEC	PRP, WP	No aggregation	-	[55]
O103, O111, O121, O157O111:B4	EHEC Non-EHEC	PRP, WP	Activation and fibrinogen binding	GPIIb/III	[39]
LPS	Not mentioned	PRP WP	-	-	[58]

(-): No reported interaction consequence, PRP: Plasma Rich Platelets, WP: Washed Platelets. FcγRIIA: Fragment crystallizable gamma Receptor, αIIbβ3 or GPIIb/IIIa: integrin alpha IIb-beta-3, EHEC: Enterohemorrhagic *E. coli*, TLR-4: Toll-Like Receptors-4, MD2: myeloid differentiation 2, MyD88: Myeloid differentiation primary response 88, CD14: Cluster of Differentiation 14.

Concerning the aggregation phenomenon, studies suggest that the complement may stimulate platelet aggregation [59,60]. Other plasma proteins might enhance platelet activation and aggregation, such as PF4 [36,61,62]. As for platelet activation, the type of LPS could be another factor of variability of platelet aggregation.

Considering all these results, the platelet-*E. coli* interaction can be described as a complex one. The results of the mentioned studies cannot determine the factor(s) creating this heterogeneity of responses, but they suggest mechanisms which could help us to understand this interaction. First, most of the *E. coli* strains tested are those involved in HUS, which does not allow for a wide range of results for interpreting the difference in behavior of bacteria towards platelets and vice versa. In addition, Ståhl noted that the STEC strain has acquired virulence genes and islands associated with pathogenicity, which makes the development of HUS multifactorial [39]. Within these studies there are also differences among the protocols used. They differ in the preparation of platelets, bacterial concentration, platelet concentration, platelet–bacteria ratio and incubation time, especially as it is known that there is an interindividual variability of platelets that requires a normalization step for the number of platelets.

Platelets are also known for their inhibitory effect against microorganisms [4,63]. They can be included in the defense by chemotaxis by appealing to immune cells or directly by secreting antimicrobial molecules, called PMPs (platelet microbicidal peptides). Among these molecules, the most described are the following: platelet-derived chemokines, also known as kinocidins (e.g., CXCL4, CXCL7 (also known as PBP) and CCL5); defensins (e.g., human β-defensin 2 (BD2)); thymosin β4 (Tβ4); and derivative antimicrobial peptides (e.g., fibrinopeptide A or fibrinopeptide B and thrombocidins (which are proteolytic derivatives of CXCL7)) [64,65,66].

While studies have shown that platelets have a bactericidal effect against *Staphylococcus aureus* due to the antimicrobial molecules secreted as a result of platelet activation [67], this inhibitory effect on Gram-negative bacteria, in particular *E. coli*, is poorly described. The few available data (Table 2) show that the bactericidal effect on *E. coli* could depend on many factors, such as the strain tested and the platelet–bacteria ratio [54,68]. Indeed, the lack of consensus regarding a standard plasma rich platelet (PRP) preparation impairs any comparisons of the effectiveness of the inhibitory effect obtained by different research groups. Another issue is the inclusion or not of leukocytes, as PRP with leukocytes (L-PRP) presents different biologic activity, which could modify inhibitory activity [69].

Tang et al. identified seven human platelet antimicrobial proteins (HPAPs): fibrinopeptide A and B, thymosin beta 4, platelet basic protein, connective tissue-activating protein 3, RANTES and PF 4, which have antibacterial properties acting against *E. coli* ML-35 in a pH and concentration-dependent manner [71]. *E. coli* ATCC11303 was also mentioned to be inhibited by PRP, while re-incubation of PRP with anti-hBD-2 antibody (human beta defensin) revealed a significant decrease of antimicrobial activity [70].

Palankar et al. showed that platelets had direct FcγRIIA-mediated antimicrobial effects and also bridged innate and adaptive immunity via chemokine PF4. Platelets were able to kill two *E. coli* K12-derived (LPS mutant) strains in a PF4-dependent manner. However, this effect was less effective against the K12 wild-type strain. The bactericidal effect of platelets would be dependent on the structure of LPS [62]. Platelets appear to be able to discriminate between different forms of LPS. It has been shown that in vitro stimulation of platelets with LPS from *E. coli* O111 or *S. minnesota*, which are, respectively, smooth and rough types of LPS, differentially induces the release of immunomodulatory molecules and therefore a differential secretion of IL-6, TNF-α and IL-8 by peripheral blood mononuclear cells [72]. Different preparations of L-PRP tested on *E. coli* ESBL-ATCC 35218 and ATCC 25922 show no inhibitory activity on the growth of these two strains [69]. This result is in agreement with the work of Ketter et al., who showed that platelets do not inhibit the growth of *E. coli* ATCC 25922 but conversely promote it [73] (Table 2). So, the stimulation of platelets leading to the secretion of antibacterial molecules is also highly variable and dependent on several factors at the bacterial and platelet level.

## 4. Discussion

**Figure 2:**
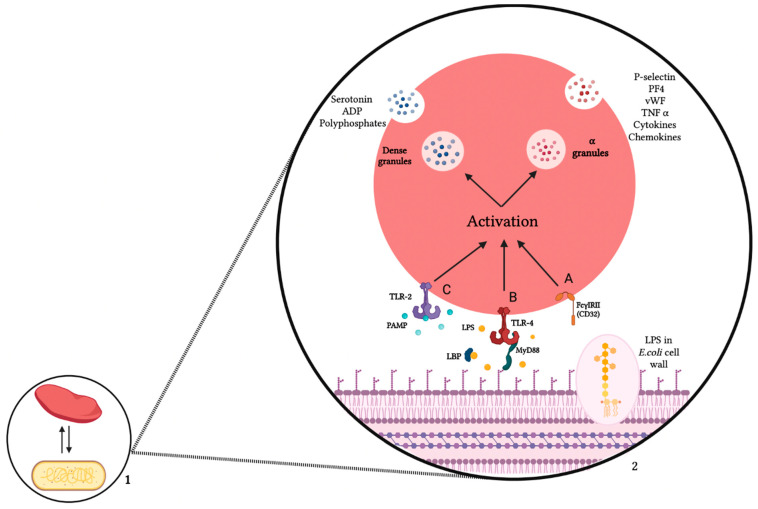
Interaction mechanisms between *E. coli* and platelets. (1) Two-way interaction between platelets and *E. coli*. (2) Mechanisms of interaction, (A) interaction via the FcγRIIA; (B) interaction via the binding between LPS and TLR4 with the intervention of MyD88 and LBP; (C) interaction with the involvement of the TLR2/PAMP complex. These three interaction mechanisms lead to platelet activation and subsequent release of alpha and dense granule contents. Created with BioRender.com, accessed on 15 February 2022. ADP: Adenosine diphosphate, PF4: Platelet Factor 4, vWF: von Willebrand factor, TNF: *Tumor Necrosis Factor*, TLR: Toll-Like Receptor, PAMP: pathogen-associated molecular pattern, LPS: Lipopolysaccharide, LBP: Lipopolysaccharide Binding Protein, MyD88: Myeloid differentiation primary response 88, FcγRIIA: Fragment Crystallizable gamma Receptor, *E. coli: Escherichia coli*.

After analyzing studies on platelets and *E. coli*, we noticed that this is a two-way interaction, and it is highly variable (Figure 2). The consequences of interactions between platelets and different strains of *E. coli* induce highly variable effects on platelet functions. Several hypotheses could explain these differences in platelet behavior depending on the *E. coli* strains tested. The most highlighted hypothesis is the difference in the LPS form, more specifically the structure of O-antigen. This variability is due to sugar variation present in the O-unit and the linkages within and between O-units. This structural heterogeneity could explain the strain-specific differences in interaction with platelets. The loss of the O-antigen is associated with a decrease in virulence of the strains [74]. The O-specific polysaccharide is able to interact directly with lysozyme and is able to inhibit its hydrolytic activity. The O-specific polysaccharide is therefore able to protect bacteria against exogenous lysozyme [75].

Resistance mechanisms to antibiotics, such as polymyxin E (colistin), may also be a factor in these differences in the behavior of different *E. coli* strains towards platelets. Antibacterial platelet peptides, mainly called cationic antimicrobial peptides (CAMPs), such as defensins, show similarities in structure and polarity with colistin [76,77]. The most common mechanism of colistin resistance is due to chromosomal mutation of genes or acquisition of plasmid genes responsible for a modification of lipid A of LPS [78,79,80] which is the main target of colistin and platelets. It can therefore be hypothesized that colistin-resistant strains would also exhibit resistance to CAMP, possibly through a modification of lipid A. Resistance to CAMPs has been demonstrated in *Haemophilus ducreyi* and *Campylobacter jejuni*, two Gram-negative bacteria producing a phosphoethanolamine transferase that is the source of polymyxin resistance [76,81].

Involved in important pathologies, *E. coli* and its effect on platelets must be further studied by testing several strains in order to understand at the molecular level the mechanisms of activation, aggregation and release of antimicrobial molecules, especially since there are contradictory results even in studies on the same strain. The involvement of multiple platelet receptors must also be highlighted in order to be able to target and inhibit the factors leading to the development of *E. coli* pathologies. Given the significant role of FcγRIIa in platelet aggregation induced by Gram-positive and Gram-negative bacteria, FcγRIIa could be an interesting drug target for conditions such as sepsis.

## Figures and Tables

**Table 2:** Summary of platelet inhibitory effect on *E. coli* strains.

*E. coli* Strain	Strain Origin	Platelet Form	Growth Inhibition Result	Factor Involved	Reference
ATCC 11303	Laboratory	PRP	+	hBD-2	[70]
ATCC 35218 ATCC 25922	Laboratory	L-PRP	−	-	[69]
ML35	Laboratory	Purified peptides from WP	+	pH = 5.5 Peptides concentration	[71]
K-12 WT	Laboratory	WP	−	-	[62]
KPM53 KPM121	Laboratory	WP	+	FcγRIIA and chemokine PF4	[62]

(+): Bacterial growth inhibition reported, (-): No growth inhibition reported, PRP: Plasma Rich Platelets, hBD-2: human Beta Defensin-2, L-PRP: leukocytes and plasma rich platelets, WP: Washed Platelets, FcγRIIA: Fragment crystallizable gamma Receptor, PF4: platelet factor 4.
